# Enhancing the Fatigue Properties of Rigid Polyurethane Foam by Dissipating the Mechanical Energy of Rubber Powder

**DOI:** 10.3390/polym17050705

**Published:** 2025-03-06

**Authors:** Jinlong Ju, Nana Yang, Yifei Zhang, Lei Yu, Guolu Ma, Wenhua Wu

**Affiliations:** 1 College of Shipbuilding Engineering, Harbin Engineering University, Harbin 150001, China; 2 Aerospace Technology Institute, China Aerodynamics Research and Development Center, Mianyang 621000, China; 3 Key Laboratory of Testing Technology for Manufacturing Process of Ministry of Education, Southwest University of Science and Technology, Mianyang 621010, China

**Keywords:** rigid polyurethane foam, fatigue behavior, rubber powder particles, compression, energy dissipation

## Abstract

Rigid polyurethane-based foam is an ideal choice for sandwich-panel-filling materials due to its high strength, low thermal conductivity, high adhesion, and high chemical resistivity. Since sandwich panel materials often face cyclic mechanical loads during their service, it is significant to study the design methods of fatigue-resistant rigid polyurethane foam and its fatigue failure mechanism to improve the performance of sandwich-panel-filling materials. In this study, a fatigue-resistant rubber powder/polyurethane composite material was prepared by introducing rubber powder, and its fatigue failure mechanism was systematically studied. The static mechanical test results indicate that with the introduction of 20% rubber powder, the compressive strength (at 85% strain) increased to 588 kPa. Additionally, thanks to the excellent energy absorption and dissipation properties of rubber powder, it can effectively dissipate mechanical energy during cyclic loading. The fatigue test results show that after the introduction of rubber powder, the fatigue life of the polyurethane foam material increases from 10,258 cycles (for PU, stress ratio 0.6) to 45,987 cycles (for 20R-PU, stress ratio 0.6). This study not only proves the fact that rubber powder can improve the fatigue performance of foam materials but also provides a potential option for the design of high-performance filling materials.

## 1. Introduction

Polyurethane foam is a porous material formed by the polymerization of polyols and isocyanates [1,2]. By adjusting the crosslinking density, polyurethane foams (rigid polyurethane foam and soft polyurethane foam) with different mechanical properties can be obtained [3]. Rigid polyurethane foam is considered to be an ideal material for sandwich panels due to its high strength and easy processing [4] and its wide range of applications in the automotive industry, including textiles, biomedicine, and construction [5,6]. For example, rigid foam has lower CO_2_ emissions, and lighter weight than traditional materials and is widely used in the automotive field [3]. It is worth noting that compressive load is an important load in the service life of rigid polyurethane foam-filling materials. The compression results of rigid polyurethane foam under random vibrations show that rigid polyurethane foam can effectively absorb energy [7]. Tuwair et al. evaluated the effect of the geometry of polyurethane foam on its compression performance and demonstrated its engineering and economic feasibility [8]. In addition to the evaluation of static mechanical properties, some studies have also examined the fatigue failure mechanism of polyurethane foam under a cyclic load [9]. In addition, related studies have found that the stiffness degradation of polyurethane foam during fatigue life follows a logarithmic trend [10]. Although these studies have effectively analyzed the static/dynamic mechanical properties of polyurethane foam, the design of fatigue-resistant polyurethane foam remains a challenge.

Generally, the failure process of polyurethane foam materials under fatigue load mainly includes two processes. First, with the increase in strain, local shear bands appear on the specific surface of polyurethane foam. With the increase in the number of cycles, these shear bands continue to expand and reduce the bearing capacity of polyurethane foam. Second, with the significant increase in strain, the pores in polyurethane foam collapse significantly [9]. To improve the fatigue performance of polyurethane foam materials, current research has proposed methods, including the introduction of granular and fibrous reinforcement materials. For example, Schäfer et al. used fiber-reinforced rigid polyurethane foam materials with three-dimensional continuous structures. The fatigue test results showed that the introduction of continuous fiber networks can effectively dissipate mechanical energy and prevent crack propagation [11]. In addition to the fiber reinforcement method, Song et al. also introduced automotive shredder residue (ASR) into polyurethane foam. Thanks to the good combination of SSR and polyurethane foam, the composite polyurethane foam material showed excellent fatigue resistance [12]. It is worth noting that whether fibrous or granular reinforcement materials are introduced, enhancing the energy dissipation capacity of composite polyurethane foam materials under cyclic load is the key to improving their fatigue performance.

**Figure 1:**
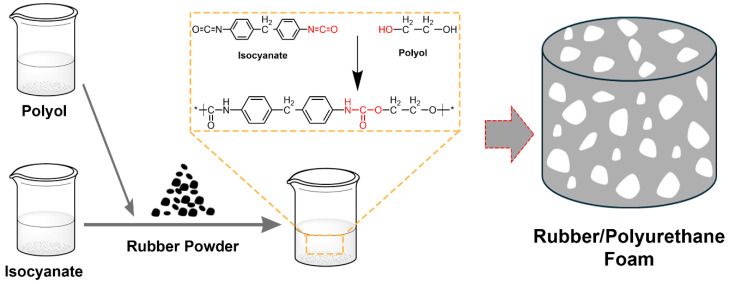
The design of the rubber/PU composite.

Rubber powder materials obtained by crushing waste rubber have attracted widespread attention and application due to their excellent mechanical properties, good energy absorption characteristics, and low cost. In the field of cement concrete, rubber powder, as an excellent toughening material [13], is widely used to improve the durability, shock absorption, friction resistance, and frost resistance of cement-based materials [14,15]. In addition to its application in cement-based materials, rubber powder also shows excellent performance and good application prospects in plywood-filling materials [16] and sound-absorbing materials [17]. Based on the excellent properties of rubber powder in energy absorption and energy dissipation, this study introduces rubber powder into polyurethane foam (Figure 1). The excellent energy dissipation of rubber powder is used to improve the fatigue performance of polyurethane foam. Modern analytical techniques are employed to analyze the influence of rubber powder on the fatigue properties of PU foam, as well as the structural evolution process and energy dissipation mechanism of rubber powder/polyurethane composite foam materials. This study provides theoretical guidance for the design and preparation of fatigue-resistant foam.

## 2. Materials and Methods

### 2.1. Materials

Toluene diisocyanate and ethylene glycol were purchased from Shanghai Aladdin Biochemical Technology Co., Ltd. (Shanghai, China). Polyester polyol was provided by Jiaxin New Materials Co., Ltd., Dongguan City, Guangdong, China. The rubber powder (60 mesh, obtained from crushed waste tires) used in this study was obtained from Shaanxi Hongrui Rubber Products Co., Ltd. (Xi’an, China).

### 2.2. Fabrication of the PU Foam

The preparation method of polyurethane-based foam refers to previous studies [4]. In short, first, a certain amount of rubber powder is mixed with toluene diisocyanate and stirred thoroughly to obtain dispersion A. Additives such as initiators are dissolved in polyester polyols and stirred thoroughly to obtain solution B. Dispersion A and solution B are then mixed and mechanical stirrer is used to quickly stir them to obtain a mixed solution C. Next, the mixed solution C is quickly transferred into a specific mold to complete the polymerization reaction. The specific chemical components are shown in Table 1.

### 2.3. Characterizations

The microstructure of the composite foam was analyzed by scanning electron microscopy (SEM, Gemini 300, ZEISS, Oberkochen, Germany). Before testing, the foam material was cut along the cross-section with a sharp knife, and gold was sprayed on the cross-section to enhance its conductivity.

### 2.4. Mechanical Properties Test

The mechanical properties of the foam material were tested by a universal mechanical testing machine (CMT6103, Mester, Shenzhen, China). The tensile and compressive properties were tested according to ASTM D3574E [18] and ASTM C365/365M [19], respectively.

**Figure 2:**
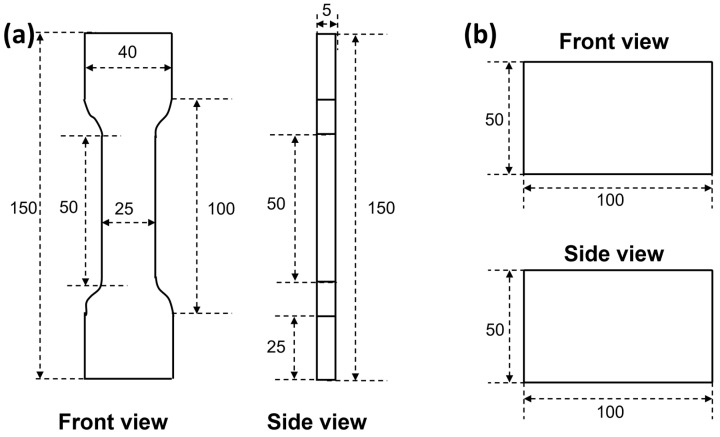
Specimens for (**a**) tensile test and (**b**) compression test.

Tensile test: First, the composite foam material was processed into a dumbbell shape (geometric dimensions are shown in Figure 2a) using a custom model. The tensile sample (length/thickness ratio 5, thickness 25 mm) was fixed with an automatic fixture, and the tensile test was carried out at a loading rate of 500 mm/min. The stress–strain curve of the sample was recorded, and 5 parallel experiments were performed for each sample.

Compressive test: First, the composite foam material was processed into a block with a size of 100 mm × 100 mm × 50 mm (Figure 2b). Then, the compressed sample was placed on the sample table of an electronic universal mechanical testing machine, a load was applied to the composite foam material at a loading rate of 0.5 mm/min, and the stress–strain curve of the compression process was recorded.

### 2.5. Fatigue Performance Test of PU Foam

The fatigue test of polyurethane foam was carried out according to previous research [9]. The fatigue test was carried out on a Shimadzu fatigue testing machine according to ASTM D1621 [20]. First, the maximum stress of the foam material was obtained according to the compression test (the stress-controlled mode, with continuous compression loads), and then the stress ratios of 0.85, 0.75, 0.65, 0.6, and 0.5 were selected for fatigue testing, and the test frequency was 3 Hz. The samples were taken out after 100, 1000, and 10,000 cycles of compression, and the evolution of the microstructure of the composite foam material was observed using a scanning electron microscope.

## 3. Results

### 3.1. Microstructure of Composite Foams

**Figure 3:**
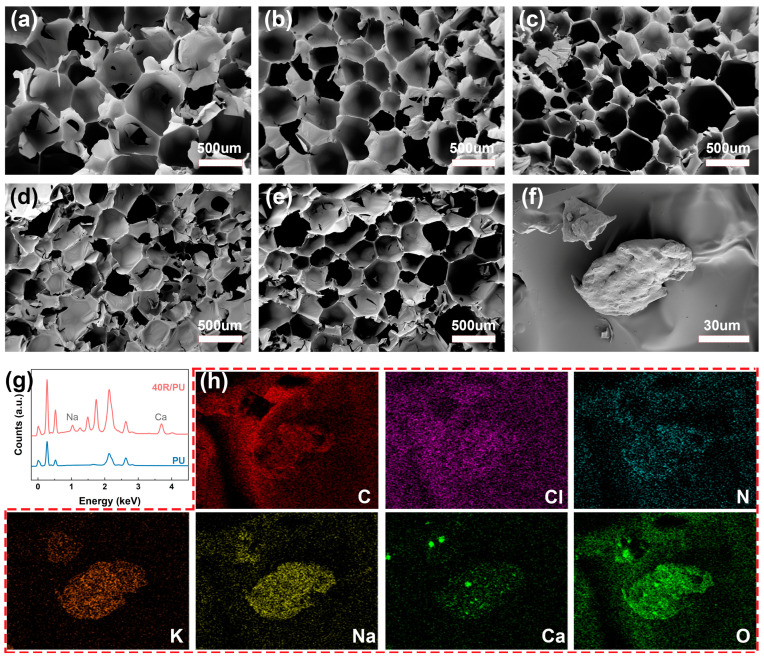
The microstructure of the (**a**) PU; (**b**) 10R-PU; (**c**) 20R-PU; (**d**) 30R-PU; and (**e**) 40R-PU rubber/PU composite. (**f**) The typical structure of rubber in 40R-PU foam; (**g**,**h**) the electron energy scattering spectrum and elemental mapping of 40R-PU.

The microstructure of the rubber powder/polyurethane foam was also observed by scanning electron microscopy. It is noteworthy that the addition of a small amount (≤20%) of rubber powder did not significantly change the porous structure of the polyurethane foam. Both 10R-PU and 20R-PU exhibit a pore structure similar to that of PU, except that the pores of the polyurethane foam become denser, and the pore size decreases from 300 to 600 μm (PU) to 200–400 μm (Figure 3a–c). When the amount of rubber powder added is greater than 30%, the uniformity of the pores decreases significantly, and obvious particles can be observed on the pore walls (Figure 3d–f).

In addition to the changes in the microstructure, characteristic spectral lines of Na, K, and Ca elements can be observed from the electron energy scattering spectrum (Figure 3g). It can also be found in the elemental mapping (Figure 3h) that the C, N, Cl, and O elements are highly coincident with the base material of the foam, and the Na, K, and Ca elements are highly coincident with the particles. Polyurethane foam materials are usually obtained by the polymerization of isocyanates and polyols [21], so Na, K, and Ca mainly come from rubber powder particles. These results indicate that rubber powder/polyurethane composite foams were successfully prepared by in situ polymerization.

**Figure 4:**
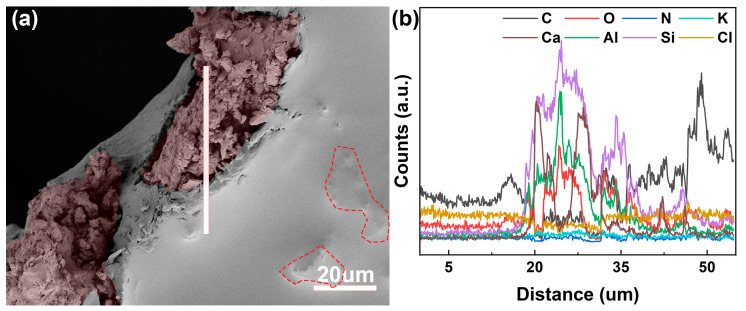
(**a**) The microstructure and (**b**) element distribution information of the rubber/PU interface, the red marked part is rubber particles.

In addition to the microstructure of rubber powder/polyurethane composites (PU, 10R-PU, 20R-PU, 30R-PU, and 40R-PU), the interface structure of rubber powder/polyurethane was also analyzed. As can be seen from Figure 4a, the rubber powder particles are almost completely embedded in the polyurethane. More information about the distribution of elements at the polyurethane/rubber powder interface is analyzed by electron energy scattering spectrum (the line-scan mode) (Figure 4b). The change pattern in the electron energy scattering spectrum in the selected area shows that in the selected area of polyurethane (0–18 μm), its elements are mainly C, N, O, and Cl, which is consistent with the element distribution of the original PU (Table 2). When entering the rubber particle area (18–54 μm), it can be clearly observed that the technology of the characteristic spectral lines of Si, Al, and Ca elements increases rapidly (Figure 4b), indicating that there is a strong interface performance between the rubber powder particles and polyurethane. Generally, the increase in the bonding performance of the particle/organic material interface is conducive to the dissipation of external energy and improves the mechanical properties of the composite material.

### 3.2. Static Mechanical Properties of Composite Foam Materials

**Figure 5:**
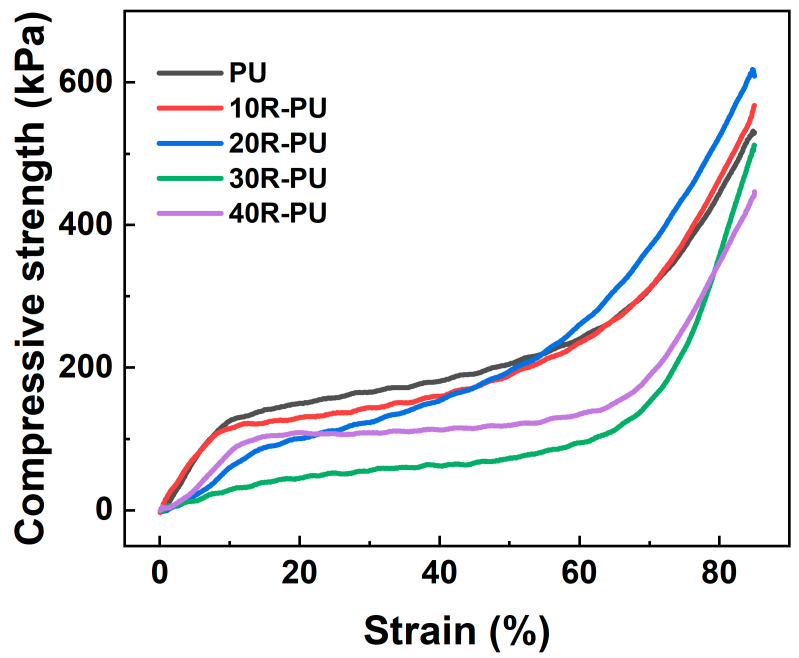
Compressive curve for PU and rubber/PU composite foams.

Mechanical properties are the key to using foam materials as filling materials. To explore the influence of rubber powder on foam materials, the mechanical properties of PU and rubber powder/polyurethane foam were evaluated by a universal mechanical testing machine. From the compression curve (Figure 5), it can be found that the compression process of PU foam can be clearly divided into the elastic zone (strain rate 0–10.9%), the platform zone (strain rate 10.9–59.6%), and the densification zone (strain rate > 59.6%) [22]. Generally, in the elastic zone, the pore structure in the foam material undergoes bending or stretching deformation under external action. Since the deformation is reversible, its shape returns to its original level after the external load is removed [23]. In the platform zone, since the foam usually undergoes irreversible structural damage, the destruction of the foam pore structure is the main way to dissipate mechanical force, so its stress changes minimally [24]. Since the pore structure of the foam material is significantly damaged in the platform zone, the pore wall structure of the foam material is in direct contact with stress [25]. Therefore, in the densification zone, stress increases significantly with strain.

**Figure 6:**
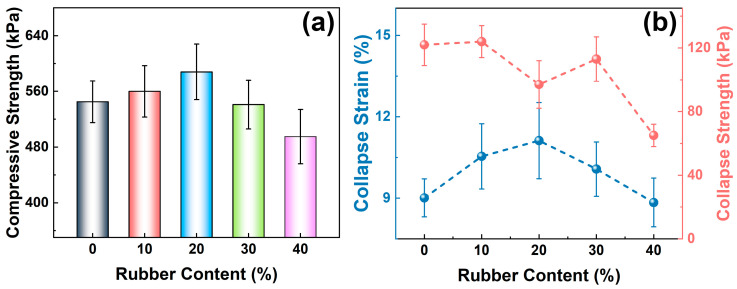
(**a**) Compressive strength and (**b**) collapse strain/strength for PU and rubber/PU composite foams.

Figure 6a shows the compressive strength of the composite foam (strain rate 85%). The compressive strength of pure PU is 545 kPa, and the compressive strength of rubber powder/polyurethane foam is related to the content of rubber powder. When a small amount of rubber powder (<20%) is added, the compressive strength increases with the rubber powder content to 560 kPa (10R-PU) and 588 kPa (20R-PU). As the rubber powder content continues to increase, the compressive strength decreases to 541 kPa and 495 kPa, respectively. These results show that the introduction of an appropriate amount of rubber powder can effectively improve the mechanical properties of polyurethane foam.

Collapse behavior is one of the important parameters of foam materials. From the collapse strength versus rubber powder content curve (Figure 6b), it is clear that the collapse strength of the rubber powder/polyurethane composite foam decreases from 122 kPa (PU) to 113 kPa (30R-PU) and 65 kPa (40R-PU) with the increase in the rubber powder content. The difference is that its collapse strain rate increases first and then decreases with the rubber powder content. 20R-PU exhibits the highest collapse strain of 11.1%, while PU (9.0%) and 40R-PU (8.8%) have lower strain rates. These results indicate that the introduction of appropriate rubber powder can effectively improve the toughness of rigid polyurethane foam.

The energy consumption during rubber powder deformation should be the main reason for the decrease in its collapse strength. Rubber powder shows higher deformation capacity and can dissipate external mechanical energy through deformation under an external load, thus improving the anti-deformation ability of the composite material. Thanks to its excellent performance, rubber powder is widely used as a filling material to improve the dynamic mechanical properties of cement-based and polymer-based materials [26]. However, due to the high density of rubber powder, a small amount of rubber powder is not enough to affect the continuity of the polyurethane matrix. Therefore, its collapse strength first increases (≤20%) and then decreases (>20%) with the rubber powder content. In addition, after a small amount of rubber powder (≤20%) is added, the foam material exhibits more uniform pores. Uniform pores can help reduce stress concentration during external loading. Therefore, 20R-PU exhibits a large collapse strain.

**Figure 7:**
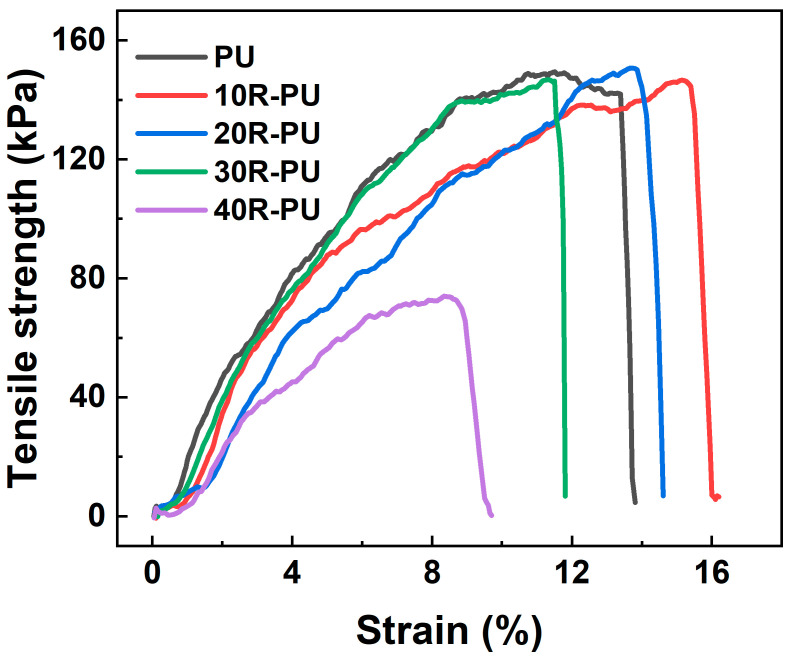
Tensile curve for PU and rubber/PU composite foams.

**Figure 8:**
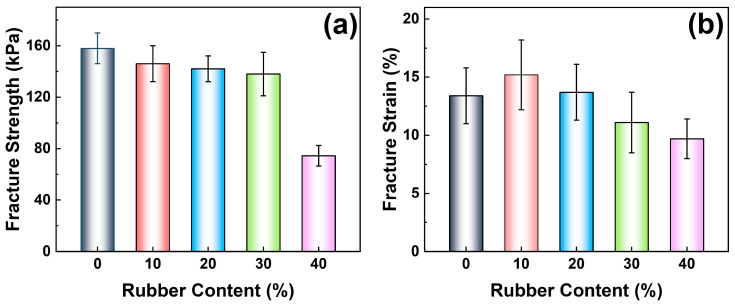
(**a**) Fracture strength, and (**b**) fracture strain for PU and rubber/PU composite foams during tensile test.

In addition to the compression properties, the tensile properties of the composite foam materials are also analyzed. Figure 7 shows the typical tensile curves of PU and rubber powder/polyurethane foam materials. To further explore the influence of rubber powder on the tensile properties of polyurethane foam, Figure 8a,b also statistically shows the fracture strength and fracture strain of the composite foam on a tensile curve. From the fracture strength, it can be found that a small addition of the amount of rubber powder does not bring a significant reduction to the polyurethane-based foam. Among them, the fracture strengths of PU, 10R-PU, 20R-PU, 30R-PU, and 40R-PU are 158 kPa, 146 kPa, 142 kPa, 138 kPa, and 75 kPa, respectively.

The reduction in the fracture strength may be mainly related to the continuity of the foam network structure. The continuous polyurethane foam network structure is the main medium for resisting external tensile loads. From the scanning electron microscope, it can be found that the inside of the foam matrix is the main distribution area of rubber powder. As the content of rubber powder increases, its distribution in the continuous network structure of polyurethane increases. It is worth noting that the density of rubber powder is high, and a small amount (≤30%) of rubber powder is not enough to produce a qualitative change in the continuous structure of polyurethane foam. When the rubber powder content is higher than 30%, due to the lack of strong chemical bonding between polyurethane and rubber powder, the polyurethane/rubber powder interface becomes a source of crack initiation and expansion, accelerating the fracture of the foam material. Therefore, the elongation at the break of the polyurethane/rubber powder composite foam decreases with the increase in the rubber powder content (Figure 8b).

### 3.3. Compression Fatigue Behavior of Syntactic Foams

**Figure 9:**
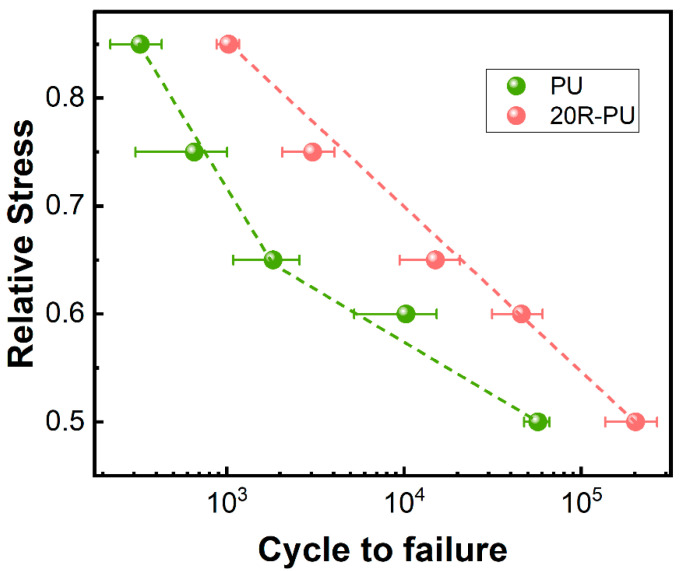
Curves depicting the ratio between relative stress and cycles to failure for the composite foams.

In addition to the static mechanical properties, the compression fatigue resistance of rubber powder/polyurethane foam was also investigated by cyclic compression tests. Figure 9 shows the fatigue life of PU and 20R-PU at different stress ratios. For pure PU foam, the corresponding fatigue lives are 325, 656, 1826, 10,258, and 56,890 at stress ratios of 0.85, 0.75, 0.65, 0.6, and 0.5, respectively. When 20% of rubber powder is added, its fatigue life is significantly increased compared with pure PU, the values of which are 1025 (stress ratio 0.85), 3054 (stress ratio 0.75), 15,025 (stress ratio 0.65), 45,987 (stress ratio 0.6) and 202,154 (stress ratio 0.5), respectively. These results show that 20R-PU has better fatigue resistance than PU.

It is worth noting that the fatigue life of PU decreases rapidly at high-stress ratios, while no similar situation is observed for 20R-PU. Generally, crack initiation and propagation are the main causes of foam failure, and crack initiation and propagation are related to the intrinsic defects of the material [27,28]. Since PU materials have no other phases, crack initiation and propagation are mainly caused by the destruction of the foam structure under compressive stress. For 20R-PU, since the rubber powder and pore structure can dissipate external mechanical energy at the same time, no obvious trend of rapid decrease in fatigue life is observed, even at high-stress ratios. These results once again prove that the addition of rubber powder can improve the fatigue performance of foam materials by dissipating energy.

**Figure 10:**
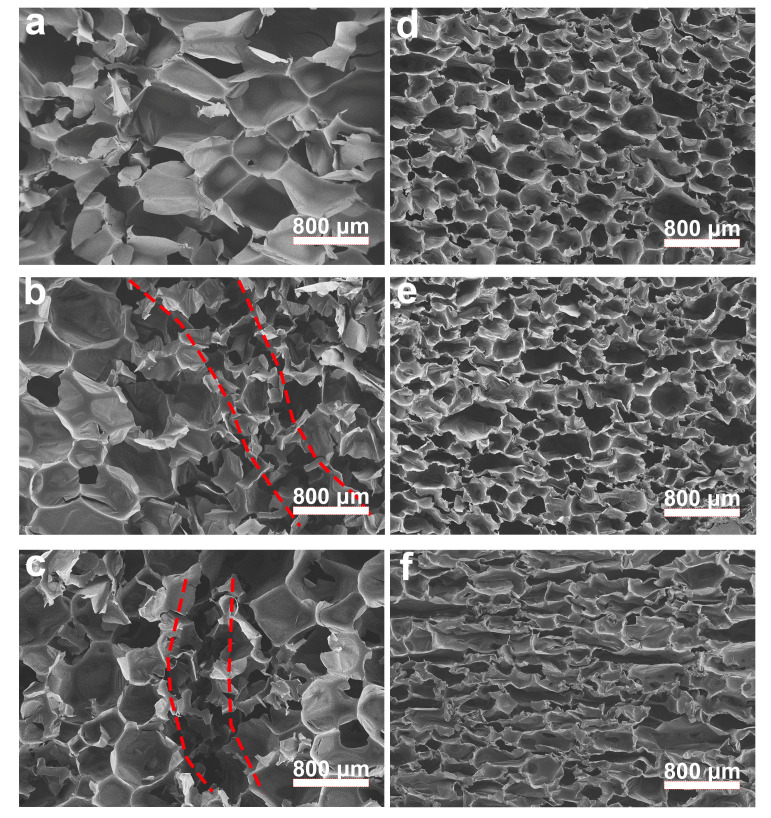
Fatigue fracture interface of the rubber/PU composite (Relative stress 0.6). The microstructure of PU with various load cycles: (**a**) ×100, (**b**) ×1000, and (**c**) ×10,000 (the crack area is between the red lines). The microstructure of 20R-PU with various load cycles: (**d**) ×100, (**e**) ×1000, and (**f**) ×10,000.

To explore the mechanism of composite foam material fracture caused by cyclic loading, the microstructures of PU and 20R-PU after 100, 1000, and 1000 loadings were analyzed by scanning electron microscopy. For the PU foam, there was no obvious change in its microstructure after 100 loadings (Figure 10a). However, after 1000 loadings, damage (the area between the red lines) could be observed inside the PU foam (Figure 10b); with the continuous accumulation of damage, obvious cracks could be observed after 10,000 cyclic compressions (Figure 10c). For 20R-PU, its microstructure did not change significantly after 100 and 1000 cyclic compressions (Figure 10d,e), and only some pore structures showed textures in the normal direction of the compression direction. As the number of cyclic compressions increased to 10,000 times (Figure 10f), almost all pores collapsed significantly along the compression direction (still no obvious crack structure was observed). These results once again show that the introduction of rubber powder can improve the toughness and fatigue resistance of PU composites by dissipating mechanical energy and preventing the initiation and propagation of cracks.

**Figure 11:**
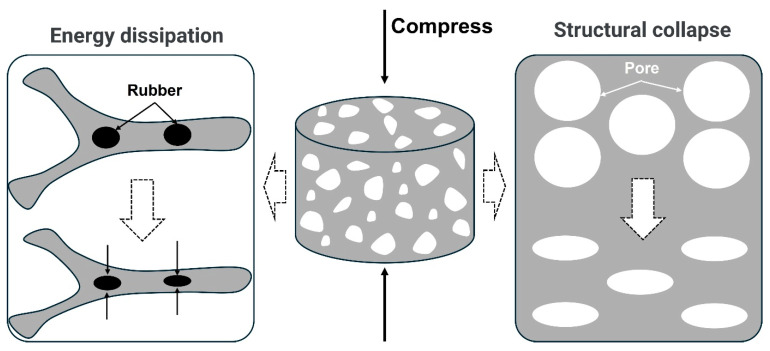
Mechanism of toughening polyurethane foam with rubber powder, the arrows point to the energy dissipation process of the composite foam material.

Based on the static/dynamic mechanical behavior and microstructural evolution of rubber powder/polyurethane foam materials, the mechanism of rubber powder enhancing the mechanical properties of polyurethane foam is summarized. As shown in Figure 11, the main reason for improving the mechanical properties of rubber powder/polyurethane composite foam materials is that rubber powder dissipates mechanical energy through the deformation and collapse of the pore structure in the foam. When the strain is small, the pore structure of polyurethane first deforms to resist the external load; as the strain continues to increase, the pore structure is not enough to resist the external load, which causes the collapse of the foam pore wall. In addition, stress is transmitted to the rubber powder particles, causing the deformation of the rubber powder. The collapse of the pore wall and the deformation of the rubber powder particles synergistically resist the external load and dissipate energy, ultimately improving the mechanical properties of the composite foam.

## 4. Conclusions

In summary, this study improved the fatigue performance of rigid polyurethane foam sandwich materials by incorporating rubber powder. Introducing a small amount of rubber powder not only makes the pore structure of the polyurethane (PU) foam denser but also dissipates mechanical energy during static and dynamic loading due to its excellent energy absorption properties. The static mechanical test results indicate that with the introduction of 20% rubber powder, the compressive strength (at 85% strain) increases to 588 kPa. Additionally, due to the regulatory properties of rubber powder on the pore structure of PU foam and its effective energy dissipation, the fatigue life of 20R-PU increases from 10,256 cycles (for PU) to 45,987 cycles (with a stress ratio of 0.6). These research findings not only demonstrate that the incorporation of rubber powder enhances the mechanical properties and fatigue resistance of PU foam but also provide theoretical guidance for the application of rigid composted PU foam.

## Figures and Tables

**Table 1:** Chemical composition of rubber powder/polyurethane composites.

Groups	Rubber (g)	Toluene Diisocyanate (mL)	Polyester Polyol (mL)
PU	0	50	50
10R-PU	10	45	45
20R-PU	20	40	40
30R-PU	30	35	35
40R-PU	40	30	30

**Table 2:** Atomic percentage of elements in syntactic polyurethane foam.

Elements	C	N	O	Cl	Na	K	Ca
PU	76.30	7.65	14.01	2.04	/	/	/
40R-PU	73.21	8.41	15.98	1.62	0.33	0.28	0.16

## Data Availability

The original contributions presented in this study are included in the article. Further inquiries can be directed to the corresponding author.
